# 

**Published:** 1915-12-11

**Authors:** 


### CONTENTS.

| | PAGE |
|---|---|
| **Professional and Popular Freedom** ... ... | 215 |
| **The Value of Percussion** ... ... ... ... | 216 |
| **Hospital and Institutional News** ... ... ... | 217 |
| A Foreword to Our Readers—The Uniform of Trained Nurses—St. George's Hospital's War Work and Needs—A "Cavell" Ward in a New Hospital—A Healthy Hospital Sunday Fund—Presentations at a Somerset V.A.D. Hospital—Medical Advertisements and the Army—A Red Cross Conference at Stockholm—The Alton Cripples' Home—The Maudsley Hospital for War Purposes—The League of Mercy—Cerebro-Spinal Fever in Australia—Trouble at Edmonton—Miss Buckingham of Birmingham—The Welsh Medical School—The Sex Question in Medical Appointments—The Day Room Revival—Infant Welfare Centres in Sussex—Child Preservation in Scotland—A Complaint against Guy's—Bradford Self-Indicted—The Portsmouth Eye and Ear Hospital—Pharmacists as "Indispensables"—Massage at Manchester—Fireguards in Hospitals—Is It a Waste of Public Money?—A Well-Merited Increase of Salary—The Extra Services of Secretaries—This Week's Drug Market. | |
| **Mind Blindness and Visual Memories** .. ... | 223 |
| How the Brain Works: The Exceptional Child. | |
| **Patriotism in British Hospitals** ... ... ... | 225 |
| Middlesex Hospital Medical School. | |
| **Acute Nephritis in the Army** ... ... ... | 226 |
| A Curious Outbreak in Flanders. | |
| **King Edward's Hospital Fund** ... ... ... | 227 |
| Modern Tendencies in Hospital Administration. | |
| **Efficient Care Committees** ... ... ... ... | 228 |
| **Voluntary Hospitals and Local Health Authorities** ... ... ... ... ... | 229 |
| What University College Hospital is Doing. | |
| **Parliament and the Profession** ... ... ... | 230 |
| **How to Prepare an Annual Report** ... ... | 231 |
| A Secretary Gives his View. | |
| **Round the World** ... ... ... ... ... | 232 |
| Fellow-Workers and the Roll of Honour. | |
| **Hospitals and the Conversion of Consols** ... | 233 |
| Pros and Cons by a Secretary. | |
| **The Editor's Letter-Box** ... ... ... ... | 233 |
| "The Population Problem." | |
| The Hospital for Sick Children. | |
| **Passing Events and Topics** ... ... ... ... | 234 |
| **Gifts and Bequests** ... ... ... ... ... | 234 |
| **Institutional Needs** ... ... ... ... ... | 234 |
| **The Institutional Worker** ... ... (Suppl.) | 1 |
| Allocation of Staff Insurances. | |
| The Choice of an X-Ray Room. | |
| Question for December. (Suppl.) | 2 |
| Enquiries and Answers. | |
| The Sick and in Need. | |
| War Matters. | |
| The Housekeeper's Department. | |

